# Covalent polyoxometalate–polyimide hybridization: multi-scale molecular engineering toward high-performance sodium-ion battery anodes

**DOI:** 10.1039/d6sc03972c

**Published:** 2026-06-15

**Authors:** Zhengyu Wei, Lingzhe Meng, Xue Qin, Wei Han, Xuelin Gong, Yiting Shi, Faheem Naseem, Wei Wei

**Affiliations:** a Department of Applied Chemistry, School of Chemistry, Xi'an Key Laboratory of Sustainable Energy Material Chemistry, Xi'an Jiaotong University Xi'an 710049 P. R. China wwei.mc@mail.xjtu.edu.cn

## Abstract

Organic electrodes suffer from poor active site accessibility, sluggish charge transport, and structural degradation upon cycling, limiting their practical application for energy storage. To address these challenges, this work elucidates a precise electronic and structural modulation strategy for polyimide (PI) *via* polyoxometalate (POM) hybridization. The key advancement lies in the multiple regulatory effects imparted by POM, enabling the construction of novel hybrid electrodes for high-performance SIBs. Specifically, the covalently anchored phosphomolybdic acid (PMo_12_) clusters disrupt π–π stacking to expose abundant active C=O sites and serve as electron-withdrawing modulators to lower the LUMO level, thereby enhancing Na^+^ uptake and transport kinetics. Simultaneously, they function as an electron-buffering reservoir to dissipate charge accumulation during discharge, preventing structural degradation of the PI matrix. This multi-scale synergy endows the PI–PMo_12_ anode with significantly improved reversible capacity, rate capability, and cycling stability, offering a promising molecular engineering strategy for developing organic–inorganic hybrid electrodes in next-generation energy storage systems.

## Abstract 2

Covalent hybridization of PMo_12_ with PI disrupts the dense π–π stacking to unlock inaccessible C=O sites and modulates the electronic structure as well as the LUMO level of PI, leading to superior sodium storage performance.

## Graphical abstract

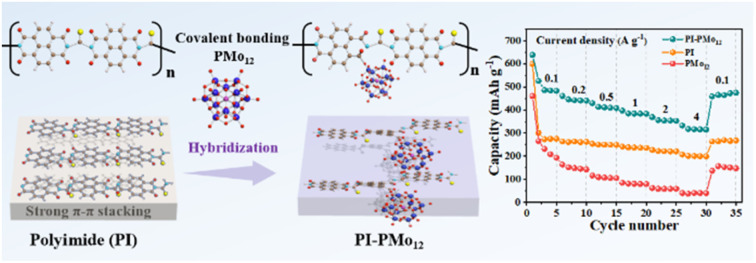

## 1. Introduction

Driven by the rapidly growing demand for large-scale energy storage, sodium-ion batteries (SIBs) have emerged as a compelling alternative to lithium-ion batteries, owing to the natural abundance and low cost of sodium resources.^1–4^ However, the practical viability of SIBs remains critically constrained by the intrinsic limitations of conventional inorganic electrode materials, such as limited specific capacity and poor cycling stability.^5^ In this context, organic electrode materials have gained considerable attention due to their structural diversity and tunability, high theoretical capacity, and environmental benignity.^6–8^ According to the redox-active moieties, organic electrode materials can be generally classified into conductive polymers, organosulfur compounds, organic radicals, and carbonyl compounds.^9^ Among these, carbonyl compounds have attracted considerable attention, primarily due to their superior redox activity and the widespread availability of precursors.^10,11^ To overcome their inherent limitations including electrolyte dissolution and poor conductivity,^12,13^ strategies including polymerization,^14^ salinization,^15^ and integration with conductive carbon supports have been widely developed.^16–18^

Polyimide (PI) electrodes exhibit distinct advantages over small-molecule carbonyl compounds, including ease of synthesis, insolubility in electrolytes, and high density of carbonyl active sites.^19,20^ Nonetheless, their electrochemical performance is severely constrained by low electrical conductivity and lack of ionic transport pathways. Extending the π-conjugation framework is a common strategy to induce electron delocalization and transport while enhancing structural integrity.^21–23^ However, this approach inevitably restricts the accessibility and utilization of active sites, thus constraining the kinetics of PI electrodes. Moreover, during deep discharge (<1.5 V), charge injection induces local charge accumulation, which induces intense electrostatic repulsion between the conjugated dianhydride moieties, resulting in structural collapse and capacity degradation.^24,25^ To date, molecular engineering approaches such as functionality grafting still face bottlenecks in resolving these issues.^26^ Benefiting from well-defined sub-nanometer architecture, rich surface chemistry, and outstanding redox properties, polyoxometalate (POM) clusters offer a versatile and effective route for multi-level modulation of PI electrodes.^27^ However, overcoming the inherent incompatibility between the PI matrix and POM mediator remains a significant challenge. Furthermore, achieving precise molecular-level covalent linkage and elucidating the underlying regulatory mechanism of POM within PI electrodes are still insufficiently explored.

In this work, we rationally designed and constructed a series of POM–PI hybrid electrodes by covalently integrating POM clusters into a PI matrix. This POM-based hybridization confers multifunctional benefits to the PI electrode in sodium storage, as corroborated by both structural evolution analyses and density functional theory (DFT) calculations. Specifically, the incorporation of PMo_12_ efficiently disrupts the compact π–π stacking of the PI backbone, exposing abundant C=O groups and boosting their redox activity and kinetics. Through robust C–O–Mo linkages and strong electron-withdrawing effects, PMo_12_ lowers the lowest unoccupied molecular orbital (LUMO) level of the hybrid electrode by modulating electron density around C=O sites, thereby markedly enhancing Na^+^ uptake. Additionally, PMo_12_ clusters act as an efficient electron buffer, mitigating charge accumulation on dianhydride units during sodiation, which alleviates structural degradation and ensures superior cycling stability. Consequently, the synergistic integration of PMo_12_ significantly accelerates charge transfer kinetics, reinforces structural integrity, and improves reversible capacity of the PI electrode. This work offers a promising strategy for multi-level molecular engineering of organic compounds and promotes the exploration of novel hybrid electrodes for high-performance SIBs.

## 2. Materials and methods

### 2.1 Materials

Naphthalene-1,4,5,8-tetracarboxylic acid dianhydride (NTCDA), thiourea, and phosphomolybdic acid hydrate (PMo_12_) were purchased from Aladdin Reagent Co. (Shanghai). The solvents including *N*,*N*-dimethylformamide (DMF), *N*-methyl-2-pyrrolidone (NMP) and ethanol were analytically pure and purchased from Tianjin Kemiou Chemical Reagent Co., Ltd. Conductive carbon (Super-P), polyvinylidene fluoride (PVDF) binder, sodium hexafluorophosphate (NaPF_6_), diethylene glycol dimethyl ether (DEGDME), aluminum foil and copper foil were purchased from Shanghai Songjing New Energy Technology Co., Ltd. All the reagents were used without further purification.

### 2.2 Syntheses of PI–PMo_12_ and PI

In a typical synthesis, equimolar amounts of NTCDA (0.268 g, 1 mmol) and thiourea (0.077 g, 1 mmol) were dissolved in DMF and stirred to mix thoroughly. Subsequently, 0.5 mmol of PMo_12_ was added, and the reaction mixture was refluxed at 175 °C under a nitrogen atmosphere for 12 hours. After cooling to room temperature, the resulting solid was isolated and washed three times with DMF and acetone to remove soluble oligomers. The insoluble solid product was then dried under vacuum at 80 °C for 12 h, followed by annealing at 300 °C for 8 h under an argon atmosphere to yield PI–PMo_12_. Using the same procedure, a series of PI–PMo_12_-*x* (*x* = 2, 3, 4, 5, 6) materials were prepared, in which the added amount of PMo_12_ was varied from 0.2 to 0.6 mmol. For comparison, PI was synthesized following an identical procedure without the addition of PMo_12_. Additionally, a physically mixed sample (denoted as PI + PMo_12_) was prepared with the same component ratio as the PI–PMo_12_ sample.

### 2.3 Structural characterization

To gain a comprehensive understanding of the structural and chemical characteristics of the samples, a range of analytical techniques were employed. Transmission electron microscopy (TEM, models JEM-F200 and JEOL) and selected area electron diffraction (SAED) were utilized to investigate the microstructure and crystallographic features. Surface morphology was further characterized using field emission scanning electron microscopy (FESEM, HT7700). Elemental distribution was visualized *via* energy-dispersive X-ray spectroscopy (EDX). Crystalline phases were identified through X-ray diffraction (XRD, Bruker D8, Germany) using Cu Kα radiation (*λ* = 0.1541 nm). The chemical states and electronic environments of the constituent elements were analyzed by X-ray photoelectron spectroscopy (XPS, Thermo Scientific), with all spectra calibrated to the C 1s peak at 284.6 eV. Functional groups and bonding information were examined using Fourier-transform infrared spectroscopy (FTIR, PE-2000, USA). Raman spectroscopy (LabRAM HR 800, 532 nm laser) was used to probe molecular vibrations and structural order.

### 2.4 Density functional theory (DFT) calculations

The calculations were performed by employing the density functional theory (DFT) as implemented in the Vienna *ab initio* simulation package (VASP). The exchange–correlation function was described using the generalized gradient approximation (GGA) parameterized by the Perdew–Burke–Ernzerhof (PBE) functional. The cut-off energy for the plane wave basis was set to 450 eV. A vacuum spacing of 15 Å was set along the *z*-direction to prevent the interaction between the slab and its periodic motif. The Monkhorst–Pack method was used for sampling the Brillouin zone with a 1 × 1 × 1 mesh. The geometry relaxation and convergence criteria for the electronic structure were 0.05 eV Å^−1^ and 1 × 10^−4^ eV, respectively. The DFT-D3 method with Grimme's scheme was employed to correct the van der Waals interactions.

### 2.5 Electrochemical measurements

To fabricate the working electrodes, the active material, Super P conductive carbon, and polyvinylidene fluoride (PVDF) binder were mixed in a weight ratio of 6 : 3 : 1 using *N*-methyl-2-pyrrolidone (NMP) as the solvent. The resulting homogeneous slurry was uniformly coated onto copper foil, followed by vacuum drying at 80 °C for 12 h. Circular electrode disks (12 mm in diameter) were then punched out, with an average mass loading of approximately 1 mg cm^−2^. For electrochemical evaluation in sodium-ion systems, 1 M NaPF_6_ in diethylene glycol dimethyl ether (DEGDME) was used as the electrolyte. A glass fiber membrane (GF/D, Whatman) served as the separator, and sodium metal was employed as the counter/reference electrode. Coin-type cells (CR2025) were assembled in an argon-filled glove box, with oxygen and moisture levels strictly controlled below 0.5 ppm. Cyclic voltammetry (CV) was conducted in the voltage range of 0.01–3.0 V using a CHI 660E electrochemical workstation (Shanghai Chenhua). Galvanostatic charge–discharge (GCD) tests were carried out between 0.01 V and 3.0 V (*vs.* Na^+^/Na) using a battery testing system (Wuhan Land Electronics Co., Ltd), and all tests were performed at ambient temperature.

For full cell construction, the cathode was prepared by mixing commercial Na_3_V_2_(PO_4_)_3_ (NVP, 1C = 117.6 mA g^−1^), Super P, and PVDF in a 7 : 2 : 1 mass ratio, dispersed in NMP to form a uniform slurry. This mixture was cast onto aluminum foil and dried at 80 °C under vacuum. Prior to assembling the full cell, the PI–PMo_12_ anode underwent two pre-sodiation cycles in half-cell configuration to compensate for the initial irreversible capacity loss. The full-cell was assembled following the same procedure as the half-cell configuration, except that NVP was used as the cathode. The full cells were constructed with a designed anode-to-cathode capacity ratio of 1.1–1.3 : 1. Electrochemical tests of the NVP//PI–PMo_12_ full cells were performed in the voltage range of 1.2–3.8 V, and current densities and specific capacities were calculated based on the active mass of the cathode.

## 3. Results and discussion

### 3.1 Covalent anchoring of PMo_12_ onto polyimide

As illustrated in Fig. S1a, NTCDA first underwent condensation polymerization with thiourea under simple high-temperature reflux to generate polyamide acid (PAA). Subsequent thermal treatment at 300 °C drove the imidization of PAA, yielding the PI. In the FTIR spectra (Fig. S2a), the characteristic C=O stretching vibration of NTCDA at 1785–1747 cm^−1^ shows a marked redshift to 1702–1680 cm^−1^ in the PI spectrum, corroborating the successful conversion of anhydride moieties to imide functionalities.^28^ Another two vibrational bands at 1345 cm^−1^ and 1265 cm^−1^ are assignable to C–N and C=S stretching modes, respectively.^29^ Further structural validation is provided by ^13^C solid-state NMR (Fig. S2b). The resonance at 163 ppm corresponds to the C=S bond of the imide ring, while signals at 167.1, 132.6 and 126.8 ppm arise from carbon atoms within the naphthalene framework.^30^ In the XRD pattern of PI, the characteristic diffraction signals of the NTCDA monomer disappear (Fig. S2c), while a new intense peak appears at 2*θ* = 28.2°. This characteristic signifies pronounced H-type stacking interactions arising from interlayer π–π conjugation.^31,32^

**Fig. 1:**
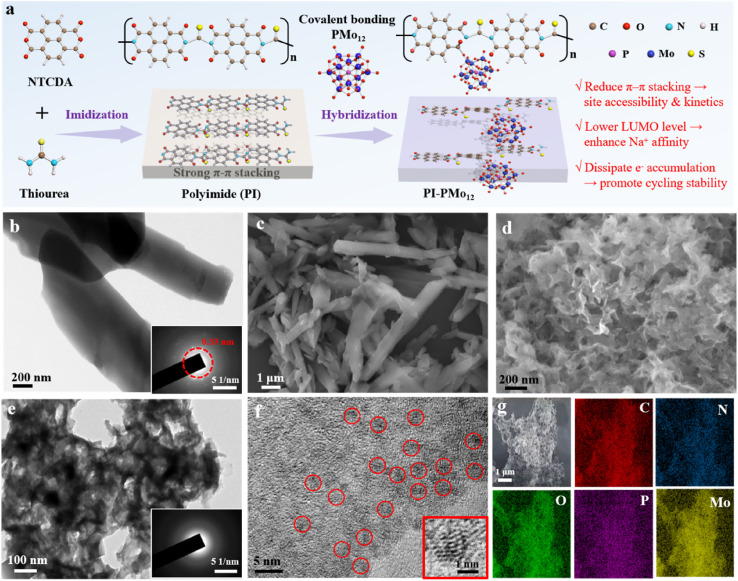
(a) Schematic of the multi-level regulation of the PI by PMo_12_ clusters; (b) TEM and (c) SEM images of PI, and the inset in (b) shows the SAED image of PI; (d) SEM and (e) TEM images of PI–PMo_12_, and the inset in (e) shows the SAED image of PI–PMo_12_; (f) HRTEM of PI–PMo_12_, red circles designate the anchored PMo_12_ clusters, and the inset shows a magnified structure of a single PMo_12_ cluster; (g) SEM and corresponding elemental mapping images of PI–PMo_12_.

Conventional PI electrodes suffer from three major limitations: (i) dense π–π stacking severely blocks carbonyl accessibility, suppressing Na^+^ storage capability; (ii) deep discharge (<1.5 V) induces charge repulsion within dianhydride units, leading to structural collapse and capacity decay; and (iii) poor electrical conductivity retards electron transport and redox kinetics. To address these limitations, POM was covalently hybridized with the PI matrix (Fig. 1a and S1b). Distinct from coordinative metal ions or small molecule modifiers, POM features a highly oxygen-rich coordination shell, a delocalized electronic structure, and reversible multi-electron redox centers, providing a versatile platform to modulate the electronic structure of active sites and facilitate interfacial charge transfer across the PI matrix. Typically, during the polycondensation process, Keggin-type PMo_12_ clusters interact with –COOH termini of PAA chains to form robust C–O–Mo covalent linkages (Fig. S3). Upon thermal imidization, the covalently anchored PMo_12_ disrupts π–π interactions and inhibits local chain packing of PI backbones through its geometrical effect. Remarkably, hybridization of PMo_12_ exerts dual electronic effects on the PI matrix: as an electron reservoir, it prevents charge-induced structural collapse; as an electron-withdrawing mediator, it lowers the LUMO level and reduces the enolization barrier. This synergy enhances the thermodynamic and kinetic performance of carbonyl redox reactions, leading to exceptional Na^+^ storage.

### 3.2 Structural characterization of the PI–PMo_12_ hybrids

The morphologies of PI and PI–PMo_12_ were characterized by SEM and TEM. As shown in Fig. 1b, c and S4a, b, PI exhibits a well-defined columnar architecture composed of π-conjugated layered stacks, with lengths ranging from 3 to 5 µm. The crystalline nature of PI is confirmed by SAED (Fig. 1b, inset), which reveals an interplanar spacing of 0.33 nm—characteristic of π–π stacking and in good agreement with the XRD analysis. Upon incorporation of PMo_12_, PI underwent a substantial morphological change (Fig. 1d, e and S4c, d), adopting a 3D-interconnected nanosheet architecture. This structural evolution is primarily ascribed to the spatial and covalent anchoring of PMo_12_ onto the polymer chains, which effectively suppresses intermolecular packing and oriented growth of PI chains. In contrast to pristine PI, SAED patterns of PI–PMo_12_ exhibit only diffuse halos (Fig. 1e, inset), indicative of an amorphous structure due to the disruption of π-conjugated stacking by PMo_12_ clusters. The high-resolution TEM (HRTEM, Fig. 1f) image demonstrates the homogeneous distribution of PMo_12_ clusters on the PI nanosheets, with individual clusters exhibiting a uniform diameter of approximately 1–1.5 nm. Furthermore, energy-dispersive X-ray (EDX) elemental mapping (Fig. 1g) confirms the uniform distribution of C, N, O, S, P, and Mo elements throughout the PI matrix.

**Fig. 2:**
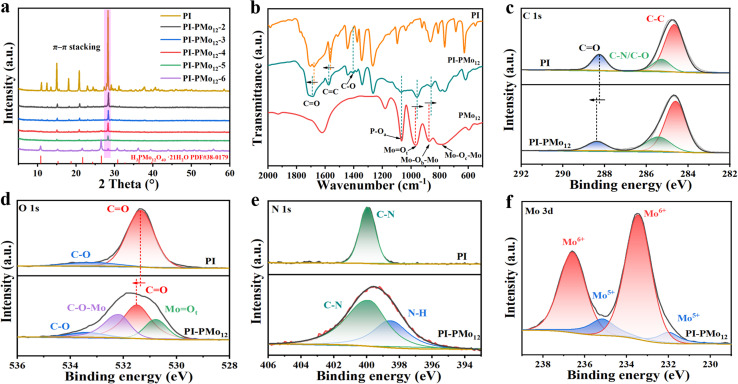
(a) XRD patterns of PI and PI–PMo_12_-*x*; (b) FT-IR spectra of PI, PI–PMo_12_ and PMo_12_; (c) C 1s, (d) O 1s and (e) N 1s XPS spectra of PI and PI–PMo_12_; (f) Mo 3d XPS spectra of PI–PMo_12_.

The hybridization of PMo_12_ significantly affected the crystalline structure of the PI matrix. As illustrated in Fig. 2a, the intensity of the π–π stacking peak gradually decreased with increasing PMo_12_ content, reaching a minimum for the PI–PMo_12_-6 sample (0.6 mmol PMo_12_ added). Concurrently, diffraction peaks characteristic of PMo_12_ (PDF #38-0179) emerged for PI–PMo_12_-6, indicating agglomeration due to excessive PMo_12_ loading. Considering that the PI–PMo_12_-5 sample achieved the optimal balance, effectively weakening π–π conjugation without inducing PMo_12_ agglomeration, it was selected as the optimal model and is hereafter simply referred to as PI–PMo_12_. The chemical structure of PI–PMo_12_ was investigated by FT-IR spectroscopy. As displayed in Fig. 2b, pure PMo_12_ exhibited characteristic peaks at 1066, 968, 873, and 786 cm^−1^, assigned to P–O_a_, Mo=O_t_, Mo–O_b_–Mo, and Mo–O_c_–Mo vibrations, respectively. In PI–PMo_12_, the Mo=O_t_ and Mo–O_b_–Mo stretching vibrations exhibited obvious red shifts compared to PMo_12_, suggesting substantial electron transfer from the PI backbone to the empty d-orbitals of Mo. Additionally, both the C=C and C=O peaks exhibited distinct blue shifts, indicating that the covalent attachment of PMo_12_ simultaneously reduces the electron density around the C=O groups and disrupts the π-conjugation stacking of the PI backbone in PI–PMo_12_.^33^ Notably, a new peak appeared at 1402 cm^−1^, which suggests the formation of C–O covalent bonds between PMo_12_ and PI.

The chemical structures of PI and PI–PMo_12_ were analyzed by XPS. The high-resolution C 1s spectra (Fig. 2c) were deconvoluted into three peaks at binding energies of 284.8, 285.4, and 288.3 eV, corresponding to C=C, C–N/C–O and C=O bonds, respectively. Compared to PI, PI–PMo_12_ exhibits a significantly enhanced C–O peak intensity, accompanied by a distinct positive shift of the C=O peak. This spectral evolution is attributed to the intermolecular C–O–Mo linkages, which effectively withdraw electron density from the carbonyl groups. The high-resolution O 1s spectra (Fig. 2d) displayed peaks at 531.4 eV, 533.2 eV and 530.7 eV, assigned to C=O, C–O and Mo=O_t_ of PMo_12_, respectively. For PI–PMo_12_, the O 1s spectra show a new peak at 532.2 eV (C–O–Mo) and a positive shift of the C=O peak, confirming the electron-withdrawing effect of PMo_12_.^34–36^ In the N 1s region (Fig. 2e), the peak at 399.9 eV corresponds to the imide C–N bond, while a new peak appeared at 398.5 eV in PI–PMo_12_, which arises from incompletely imidized-NH groups due to PMo_12_ hybridization.^37^ The Mo spectra of PI–PMo_12_ could be further resolved into Mo^6+^ and Mo^5+^ states (Fig. 2f), verifying the electronic interaction between PMo_12_ and PI. This strong electronic coupling establishes efficient charge-transfer channels, which may significantly improve the performance in SIBs.^38^ The high-resolution P 2p spectrum of PI–PMo_12_ (Fig. S5a) exhibits a spin–orbit doublet at 134.1 eV (P 2p_3/2_) and 135.0 eV (P 2p_1/2_), which is assigned to the P(v)–O in the PO_4_ tetrahedron units of PMo_12_.^39^ In the Raman spectrum (Fig. S5b), the bands observed at 967 and 984 cm^−1^ are attributed to the stretching mode vibrations of the MoO_6_ octahedron in PMo_12_ clusters.^38,40^ These results confirm the well-retained Keggin structure of PMo_12_ in the hybrid materials.

### 3.3 Electrochemical performance of PI–PMo_12_ hybrids

**Fig. 3:**
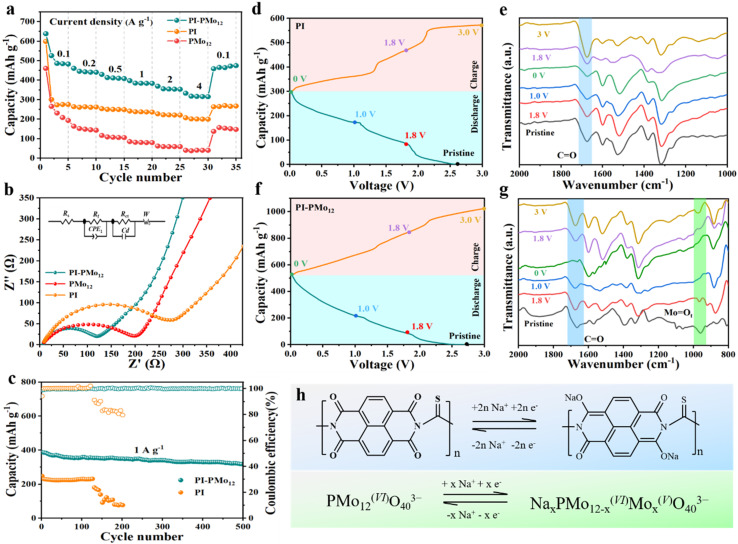
(a) Rate performance and (b) Nyquist plots of PI, PMo_12_ and PI–PMo_12_ anodes (inset shows the equivalent circuit diagram); (c) long-term cycling stability and CEs of PI–PMo_12_ and PI anodes; (d) GCD profiles and (e) *ex situ* FTIR spectra at different charge–discharge states of PI; (f) GCD profiles and (g) *ex situ* FTIR spectra at different charge–discharge states of PI–PMo_12_; (h) the proposed sodium storage mechanism for PI–PMo_12_.

The electrochemical performance of PI–PMo_12_ as an anode for SIBs was evaluated *via* half-cell testing. Fig. S6a displays the GCD profiles of PI–PMo_12_ from the 1st to the 5th cycle at a current density of 0.1 A g^−1^. The PI–PMo_12_ electrode delivers initial discharge/charge capacities of 639.5/504.8 mAh g^−1^, corresponding to a high initial coulombic efficiency (ICE) of 78.9%. In contrast to that of pristine PI (49.5%) and PMo_12_ (55.7%) (Fig. S6b and c), the markedly enhanced ICE of PI–PMo_12_ arises from the covalent integration of PMo_12_, which enables a more efficient and reversible enolization process and thereby suppresses irreversible side reactions. The PI–PMo_12_ anode also demonstrates outstanding rate capability (Fig. 3a and S6d). As the current density increased from 0.1 A g^−1^ to 4 A g^−1^, only a marginal decrease in reversible capacity was observed, suggesting favorable kinetics within the electrode. Specifically, it delivered initial discharge capacities of 526.2, 460.9, 429.6, 397.3, and 369.1 mAh g^−1^ at current densities of 0.1, 0.2, 0.5, 1, and 2 A g^−1^, respectively. Even at 4 A g^−1^, a remarkable capacity of 332.9 mAh g^−1^ was retained, surpassing the performance of pure PI, PMo_12_ and PI + PMo_12_ (Fig. S6e, f and S7a). This exceptional rate performance originates from a synergistic mechanism. First, the incorporation of PMo_12_ disrupts strong π–π conjugation among PI backbones, creating more accessible C=O active sites to boost storage capacity. Second, the robust C–O–Mo covalent bonds between PI and PMo_12_ modulate the electronic structure and local environment of the carbonyl groups, enabling efficient kinetic transport.

The ion diffusion behavior and charge transfer resistance were investigated using electrochemical impedance spectroscopy (EIS, Fig. 3b). Typically, the mid-to-high frequency region reflects the ohmic resistance (*R*_s_), solid electrolyte interphase (SEI) film resistance (*R*_f_), and charge transfer resistance (*R*_ct_) at the electrode/electrolyte interface, while the low-frequency sloping line reflects the diffusion characteristics of Na^+^. Equivalent circuit fitting revealed that the PI–PMo_12_ electrode exhibits a much lower *R*_ct_ value (103.9 Ω) compared to pure PI (239.2 Ω) and PMo_12_ (169.2 Ω). Furthermore, a steeper slope was observed in the low-frequency Warburg region, indicating enhanced Na^+^ diffusion kinetics. Galvanostatic intermittent titration technique (GITT) measurements were performed to evaluate the Na^+^ diffusion coefficient. As depicted in Fig. S8, the GITT profiles reveal an obviously reduced voltage polarization for PI–PMo_12_ relative to PI. The PI–PMo_12_ anode exhibits an average Na^+^ diffusion coefficient of 8.59 × 10^−11^ cm^2^ s^−1^, significantly higher than that of PI (1.62 × 10^−11^ cm^2^ s^−1^). The PI–PMo_12_ anode also exhibited exceptional cycling stability. At a current density of 0.2 A g^−1^, it retained a reversible capacity of 409.2 mAh g^−1^ after 100 cycles (Fig. S9a). When the current increased to 1 A g^−1^, the anode retained a stable capacity of 317.5 mAh g^−1^ over 500 cycles with CE consistently exceeding 99% (Fig. S9b). In contrast, both pristine PI and the PI + PMo_12_ electrode suffered from severe capacity decay after only 127 and 139 cycles, respectively (Fig. 3c and S7b).

*Ex situ* FTIR spectroscopy was conducted over a voltage window of 0.01–3.0 V to monitor the evolution of redox-active groups during discharge/charge. As shown in Fig. 3d and e, the C=O stretching vibration at 1680 cm^−1^ in the pristine PI gradually diminishes upon discharging and recovers upon charging, corresponding to the reversible enolization reaction of the carbonyl groups. It should be noted that a residual C=O signal persists even at 0 V, resulting from limited accessibility and reaction due to strong interchain π–π stacking. In stark contrast, the C=O vibration in PI–PMo_12_ nearly vanishes when discharged to 0 V (Fig. 3f and g). This complete conversion of carbonyl groups highlights the key role of PMo_12_ in mitigating π–π stacking, thereby unlocking a greater fraction of C=O sites for reversible Na^+^ storage. Simultaneously, the Mo=O vibration of PMo_12_ disappears during discharging and reappears upon charging, demonstrating the reversible redox process of the PMo_12_ units. Based on the aforementioned analysis, we propose a sodium storage mechanism for PI–PMo_12_ (Fig. 3h). The C=O groups on the PI backbone serve as the primary active sites, which accommodate two Na^+^ and two electrons *via* an enolization reaction, with the reverse process occurring during the charging. Meanwhile, the PMo_12_O_40_^3−^ clusters undergo a multi-electron redox reaction involving the Mo^6+^/Mo^5+^ couple, accompanied by reversible Na^+^ insertion/extraction.

### 3.4 Mechanistic insight into PMo_12_ modulation of the PI electrode

**Fig. 4:**
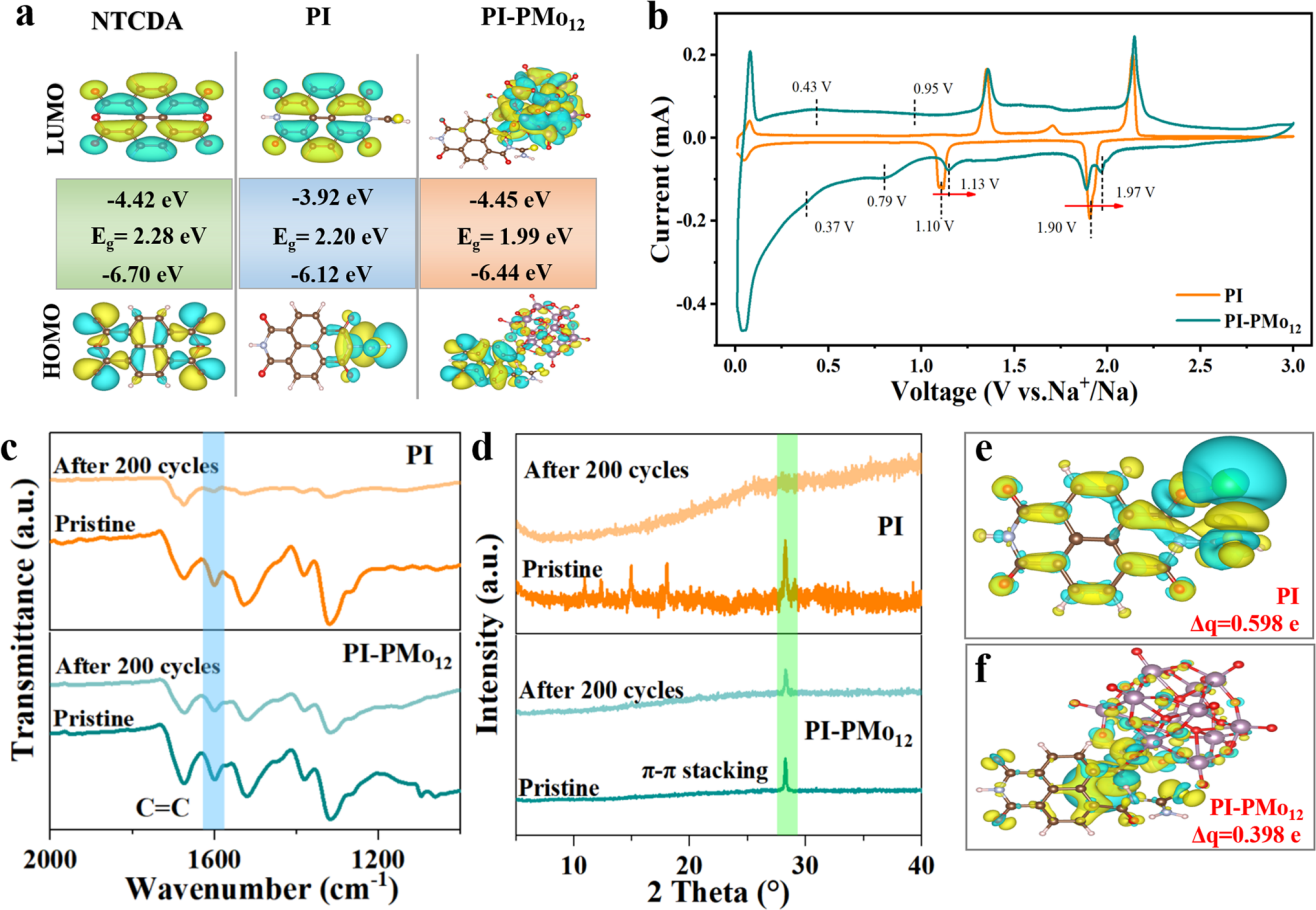
(a) Molecular structures, HOMO/LUMO energy levels and orbital distributions of NTCDA, PI and PI–PMo_12_; (b) CV curves of PI and PI–PMo_12_ anodes at a scan rate of 0.2 mV s^−1^; (c) FTIR spectra and (d) XRD patterns of PI and PI–PMo_12_ before and after 200 cycles; the differential charge density map of (e) PI and (f) PI–PMo_12_ after the sodiation process.

Compared with pristine PI, the as-constructed PI–PMo_12_ hybrid exhibits significantly enhanced electrochemical performance for SIBs. To elucidate the modulation mechanism of PMo_12_, we systematically investigated the electronic structures of NTCDA, PI and PI–PMo_12_ using DFT calculations (Fig. 4a). According to molecular orbital theory, the energy of the LUMO is directly related to electron affinity.^24^ Despite its low LUMO energy level, NTCDA is unsuitable as an electrode material due to its severe dissolution in organic liquid electrolytes. Notably, the LUMO level of PI–PMo_12_ (−4.45 eV) is significantly lower than that of pristine PI (−3.92 eV). PDOS analysis (Fig. S10a) further reveals that PMo_12_ plays a key role in reducing the LUMO of the PI backbone *via* its strong electron-withdrawing effect. The lowered LUMO level facilitates electron acquisition, thereby thermodynamically promoting the Na^+^ storage capability. We further calculated the Na^+^ adsorption energies (*E*_ads_) at three representative sites in the PI–PMo_12_ model (Fig. S10b). The PMo_12_ cluster exhibits the strongest Na^+^ adsorption affinity (site I, *E*_ads_ = −1.90 eV), which aligns with its intrinsic characteristics of ready reducibility. The carbonyl sites modified by C–O–Mo covalent bonds also exhibit strong Na^+^ adsorption capability (site II, *E*_ads_ = −1.21 eV), which is greatly enhanced compared to these unconnected C=O groups (site III, *E*_ads_ = −0.69 eV). Moreover, PI–PMo_12_ exhibits a narrower band gap (*E*_g_) between the highest occupied molecular orbital (HOMO) and LUMO compared to PI, suggesting enhanced electronic conductivity when used as an anode material for SIBs.^41^ This effect is clearly evidenced by the cyclic voltammetry (CV) measurements of the PI–PMo_12_ and PI electrodes within a voltage window of 0.01–3.0 V (Fig. 4b). The PI–PMo_12_ electrode exhibits two prominent pairs of redox peaks located at 1.13/1.35 V and 1.9/2.1 V, corresponding to the two-step enolization reaction of the carbonyl groups.^42^ Additionally, two redox couples appearing at 0.37/0.43 V and 0.79/0.95 V are associated with the reduction/oxidation of PMo_12_ (Fig. S11).^43^ Notably, PI–PMo_12_ exhibits a positive shift in its reduction peak from 1.10 V to 1.13 V relative to pristine PI, indicating that the covalently anchored PMo_12_ withdraws electron density from the carbonyl sites and thus reduces the overpotential for Na^+^ uptake. In addition, the reduction peak at 1.90 V splits into two peaks, showing a small additional peak at 1.97 V, suggesting the existence of new Na^+^ uptake sites associated with carbonyl sites covalently bonded to PMo_12_. The obvious change in the characteristic CV peaks demonstrates that electronic modulation by PMo_12_ not only facilitates the reduction of carbonyl groups but also introduces additional redox-active sites for sodium storage.

During deep discharge, negative charges accumulate on the conjugated dianhydride units of PI, generating strong electrostatic repulsion that ultimately leads to structural collapse and capacity loss (Fig. 3c). FTIR and XRD were employed to probe the structural evolution of both electrodes after 200 cycles. FTIR analysis (Fig. 4c) reveals that the C=C vibrational signal of pristine PI nearly disappears after cycling, indicating severe degradation of its conjugated dianhydride framework. In contrast, the C=C signal of PI–PMo_12_ remains clearly visible, confirming that its structure is largely preserved. XRD patterns (Fig. 4d) further corroborate this degradation, as all characteristic diffraction peaks of pristine PI vanish after 200 cycles. Conversely, PI–PMo_12_ exhibits negligible structural change and retains its π–π stacking characteristics throughout cycling. These results highlight the crucial role of PMo_12_ in preserving the structural integrity of PI. The differential charge density map reveals distinct charge redistribution after enolization (Fig. 4e and f). Pristine PI exhibits significant electron accumulation (yellow regions) around the conjugated dianhydride units. In contrast, the PI–PMo_12_ composite shows negligible charge buildup on these units, with electrons primarily accumulating around the PMo_12_ clusters. Bader charge analysis (Fig. S12a, b and Tables S1, S2) quantifies the electron accumulation, revealing that upon sodiation, 0.598e^−^ accumulate on pristine PI, whereas only 0.398e^−^ accumulate on the PI segment within the PI–PMo_12_ hybrids. Thus, PMo_12_ effectively mediates electrons during enolization, regulating charge distribution across the PI backbone and preventing structural degradation. The enhanced structural integrity of PI–PMo_12_ is further evidenced by the measured C–O^−^ bond elongation (Fig. S12c). After sodiation, pristine PI exhibits a C–O^−^ bond elongation of 0.04 Å, while that of PI–PMo_12_ is only 0.02 Å. This smaller elongation indicates less local geometric distortion of carbonyl groups upon Na^+^ insertion. As a result, after being immersed in the electrolyte solvent, pristine PI shows obvious color change due to dissociation into soluble species, whereas PI–PMo_12_ showed no visible color change, demonstrating exceptional durability (Fig. S13). This remarkable stability underscores the ability of PMo_12_ to mitigate detrimental electron accumulation around the PI backbone, thereby guaranteeing outstanding cycling stability.

### 3.5 Extended construction of PI–POM hybrids and application in full SIBs

**Fig. 5:**
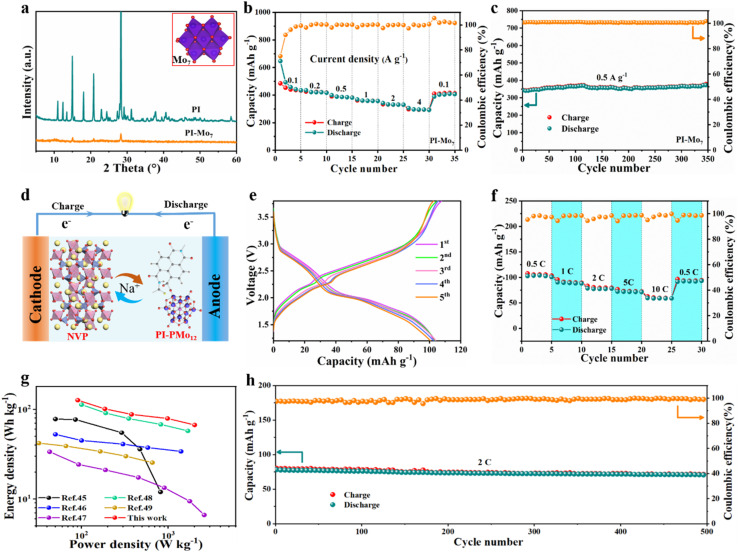
(a) XRD patterns of PI and PI–Mo_7_ (the inset shows the geometric shape of the Mo_7_ cluster); (b) rate performance, (c) cycling stability and CEs of the PI–Mo_7_ anode at 0.5 A g^−1^; (d) schematic diagram of full SIBs using PI–PMo_12_; (e) GCD profiles at 0.5C and (f) rate performance of the NVP//PI–PMo_12_ full cell; (g) Ragone plots of the NVP//PI–PMo_12_ full cell in comparison with recently reported SIB full cells;^45–49^ (h) long-term cycling stability and CEs of the NVP//PI–PMo_12_ full cell at 2C.

The incorporation of PMo_12_ simultaneously optimizes the interchain stacking of PI and the electronic structure of C=O groups, which is essential for achieving efficient Na^+^ storage and long-term cycling stability. Beyond the Keggin-type PMo_12_, this hybridization strategy can be extended to other types of POM,^44^ such as ammonium heptamolybdate (Mo_7_) and ammonium octamolybdate (Mo_8_). Similar to PI–PMo_12_, Mo_7_ and Mo_8_ can also condense with the PI matrix through their abundant surface Mo=O sites, realizing analogous modulation of electronic and structural properties of PI. The structures of resultant PI–POM hybrids were verified by FTIR and XRD. As shown in Fig. S14a and b, both PI–Mo_7_ and PI–Mo_8_ exhibit characteristic peaks near 1402 cm^−1^, corresponding to the C–O bond, indicating the covalent bonding of Mo_7_/Mo_8_ clusters onto the PI matrix. Fig. 5a and S14c reveal a significant weakening of the π–π stacking characteristic peaks in both PI–Mo_7_ and PI–Mo_8_, indicating the spatial effect of POM clusters on the conjugation of PI. TEM images (Fig. S15) reveal that both PI–Mo_7_ and PI–Mo_8_ possess a 3D-interconnected nanosheet architecture similar to that of PI–PMo_12_. HRTEM images further confirm the uniform distribution of sub-nm-sized (1–1.5 nm) Mo_7_ and Mo_8_ clusters within the PI matrix. Similarly, both PI–Mo_7_ and PI–Mo_8_ demonstrate excellent Na^+^ storage capability in terms of rate performance and cycling stability. As shown in Fig. 5b and S16a, when the current density increases from 0.1 to 4 A g^−1^, both electrodes exhibit only moderate capacity decay. At a high rate of 4 A g^−1^, they still deliver high reversible capacities exceeding 260 mAh g^−1^. The cycling performance of both electrodes at 0.5 A g^−1^ over 350 cycles is shown in Fig. 5c and S16b. Both electrodes retain high reversible capacity and CEs close to 100%, demonstrating superior reversibility of the POM-hybridized PI electrodes during Na^+^ insertion/extraction.

To assess the practicality of the PI–POM hybrids for SIBs, a full cell was assembled with NVP as the cathode and PI–PMo_12_ as the anode (Fig. 5d). The NVP cathode was first evaluated in a half-cell (Fig. S17), delivering a reversible capacity of 107.4 mAh g^−1^ at 0.1C. The NVP//PI–PMo_12_ full cell was then assembled and tested between 1.2 V and 3.8 V (Fig. 5e), with capacities normalized to the mass of NVP. At 0.5C, it achieved a high discharge capacity of 102.8 mAh g^−1^, and the nearly overlapping GCD curves in subsequent cycles confirm excellent reversibility. The full cell demonstrated outstanding rate performance (Fig. 5f and S18a), exhibiting initial discharge capacities of 102.8, 90.4, 78.5, and 72.2, 59.5 mAh g^−1^ at 0.5, 1, 2, and 5C, and 10C, respectively. When the rate was returned to 0.5C, a stable capacity of 98.2 mAh g^−1^ was recovered. Moreover, the NVP//PI–PMo_12_ full cell possesses excellent energy and power densities. The Ragone plot (Fig. 5g) reveals an energy density of 126.6 Wh kg^−1^ at a power density of 90.5 W kg^−1^, and retains 67.0 Wh kg^−1^ even at a high power density of 2010.3 W kg^−1^, outperforming recently reported NVP-based SIBs.^45–49^ The cell also exhibited outstanding cycling stability: a reversible capacity of 100.3 mAh g^−1^ was retained after 100 cycles at 0.5C (97.6% retention, Fig. S18b). Even at a higher rate of 2C, a stable capacity of 70.0 mAh g^−1^ was retained after 500 cycles, corresponding to 89.4% retention (Fig. 5h).

## 4. Conclusion

In summary, this work fundamentally elucidates a precise electronic and structural modulation strategy for organic electrodes *via* POM hybridization, in which PMo_12_ is covalently integrated into a PI matrix to create a high-performance SIB anode. The key advancement lies in the multiple regulatory effects imparted by POM. Through intermolecular C–O–Mo linkages, PMo_12_ acts as an electron-withdrawing modulator that actively lowers the LUMO level of the PI matrix, thereby enhancing its redox activity and Na^+^ affinity. Simultaneously, it functions as an electron-buffering reservoir that dissipates localized charge accumulation during discharge, preventing the structural degradation of conjugated dianhydride units. Moreover, the incorporation of PMo_12_ disrupts the excessive π–π stacking of PI chains, exposing more active C=O sites and facilitating ion accessibility. This multi-scale synergistic integration provides significantly improved reversible capacity, rate capability, and cycling stability, offering a promising molecular-level engineering strategy for developing organic–inorganic hybrid electrodes in next-generation energy storage systems.

## Author contributions

Zhengyu Wei: performed experiments, analyzed the data, and drafted the original manuscript. Lingzhe Meng: methodology. Xue Qin: conceptualization. Wei Han: visualization, methodology. Xuelin Gong: formal analysis. Yiting Shi: methodology. Faheem Naseem: resources. Wei Wei: funding acquisition, project administration, writing – review and editing, visualization.

## Conflicts of interest

The authors declare no conflict of interest.

## Supplementary Material

SC-017-D6SC03972C-s001

## Data Availability

The data that support the findings of this study are available from the corresponding author upon reasonable request. Supplementary information (SI) is available. See DOI: https://doi.org/10.1039/d6sc03972c.
