# Supplementary material for: Covalent polyoxometalate–polyimide hybridization: multi-scale molecular engineering toward high-performance sodium-ion battery anodes
Source: Chem Sci. 2026 Jun 15. Online ahead of print. doi: 10.1039/d6sc03972c (PMC13324796; doi:10.1039/d6sc03972c)
Supplement: SC-OLF-D6SC03972C-s001 [file SC-OLF-D6SC03972C-s001.pdf]

Supplementary Information (SI) for Chemical Science.
This journal is © The Royal Society of Chemistry 2026

Supplementary Information for

Covalent Polyoxometalate-Polyimide Hybridization: Multi-Scale Molecular Engineering toward High-Performance Sodium-Ion Battery Anodes

Zhengyu Wei, Lingzhe Meng, Xue Qin, Wei Han, Xuelin Gong, Yiting Shi, Faheem Naseem, Wei Wei*

Department of Applied Chemistry, School of Chemistry, Xi'an Key Laboratory of Sustainable Energy Material Chemistry, Xi'an Jiaotong University, Xi'an 710049, P. R. China

*Corresponding author:

E-mail: wwei.mc@mail.xjtu.edu.cn ORCID: 0000-0002-8357-8427

■ SUPPORTING FIGURES

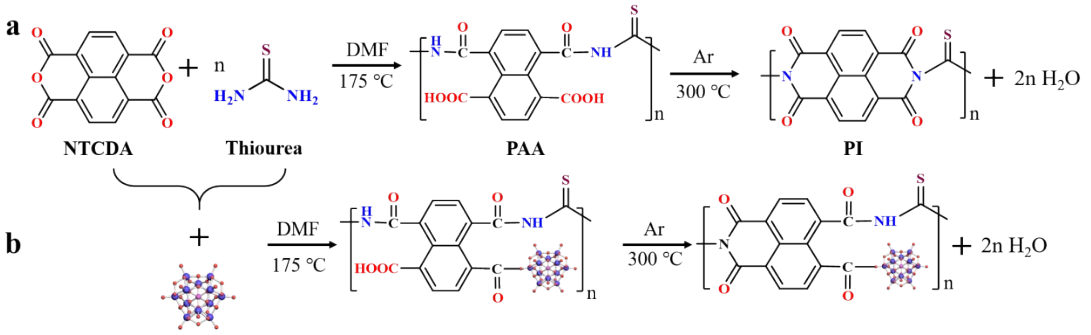

Fig. S1 Synthetic route of (a) PI and (b) PI-PMo_{12}.

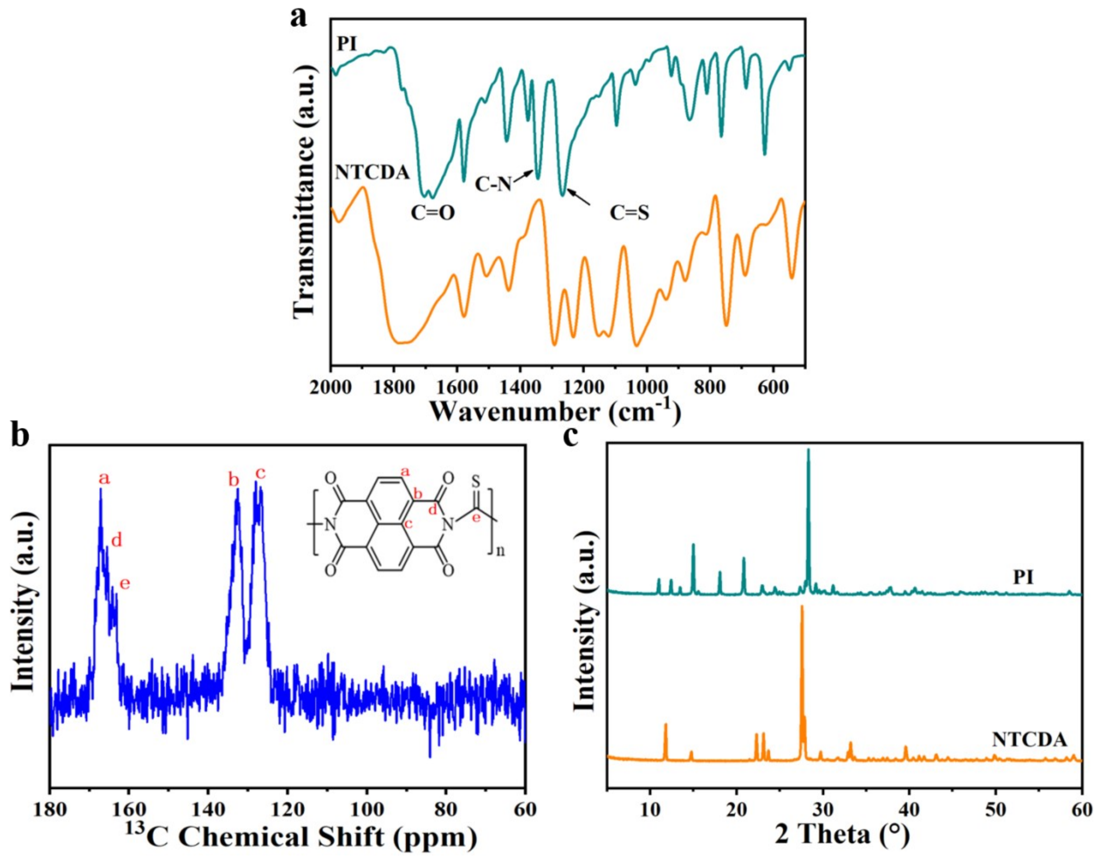

Fig. S2 (a) FTIR spectra of PI and NTCDA; (b) ^{13}C solid state NMR spectra of PI, the inset is the molecular structures of PI; (c) XRD spectra of PI and NTCDA.

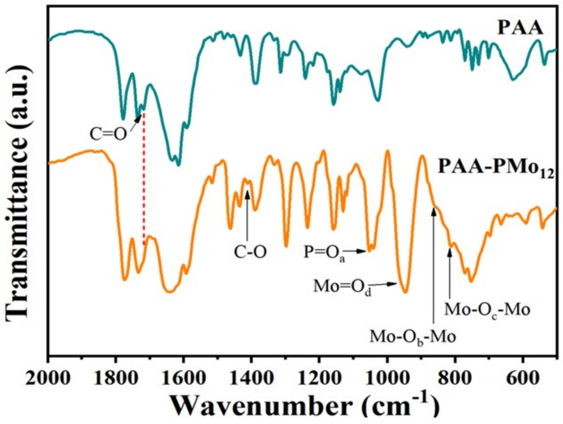

Fig. S3 FT-IR spectra of PAA and PAA-PMo_{12}.

FTIR spectroscopy confirms the covalent hybridization between PMo_{12} and PAA. Compared with the pure PAA spectrum, the 1720 cm^{-1} characteristic peak representing the C=O stretching vibration in -COOH disappears[1, 2]. Meanwhile, a new peak emerges at 1402 cm^{-1}, which is attributed to the formation of C-O bonds. Furthermore, the distinct vibrations of the PMo_{12} are clearly observed at 1053, 946, 860, and 811 cm^{-1}, assigned to the P-O_a, Mo=O_t, Mo-O_b-Mo, and Mo-O_c-Mo stretching modes, respectively[3, 4]. These spectroscopic changes collectively demonstrate that a condensation reaction occurs between PMo_{12} and the carboxyl groups of PAA and form covalent C-O-Mo linkages.

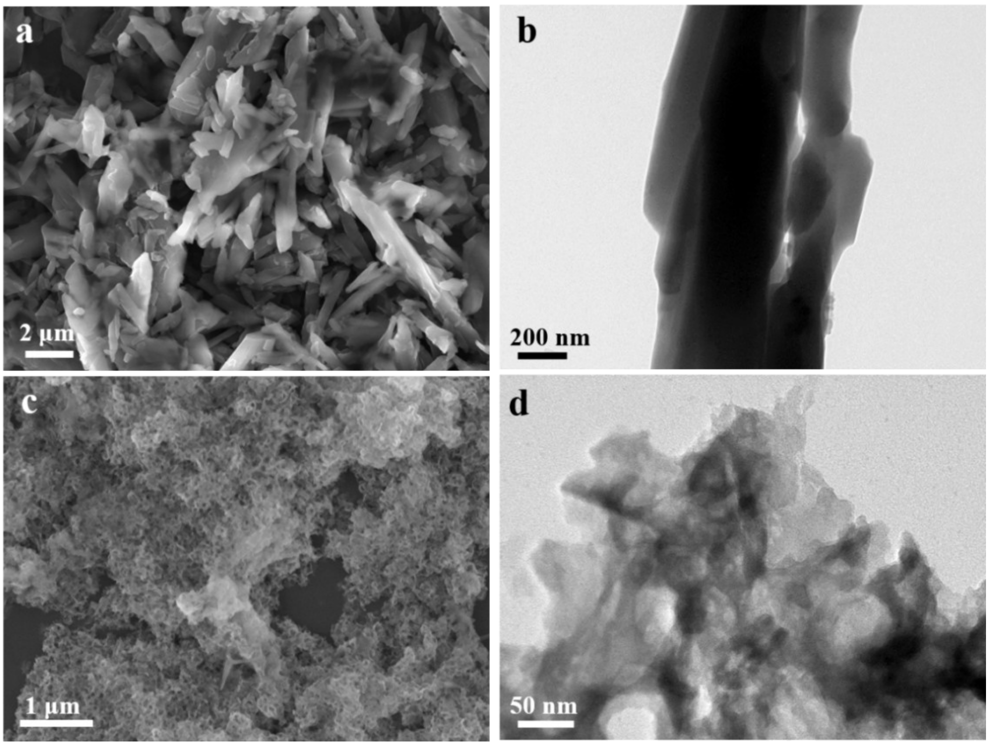

Fig. S4 (a) SEM and (b) TEM images of PI; (c) SEM and (d) TEM images of PI-PMo_{12}.

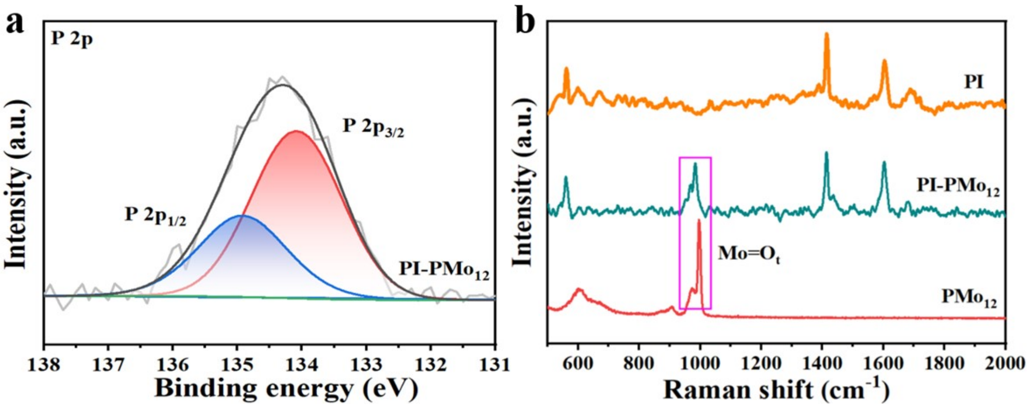

Fig. S5 (a) P 2p XPS spectra of PI-PMo_{12}; (b) Raman spectra of PI, PI-PMo_{12} and PMo_{12}.

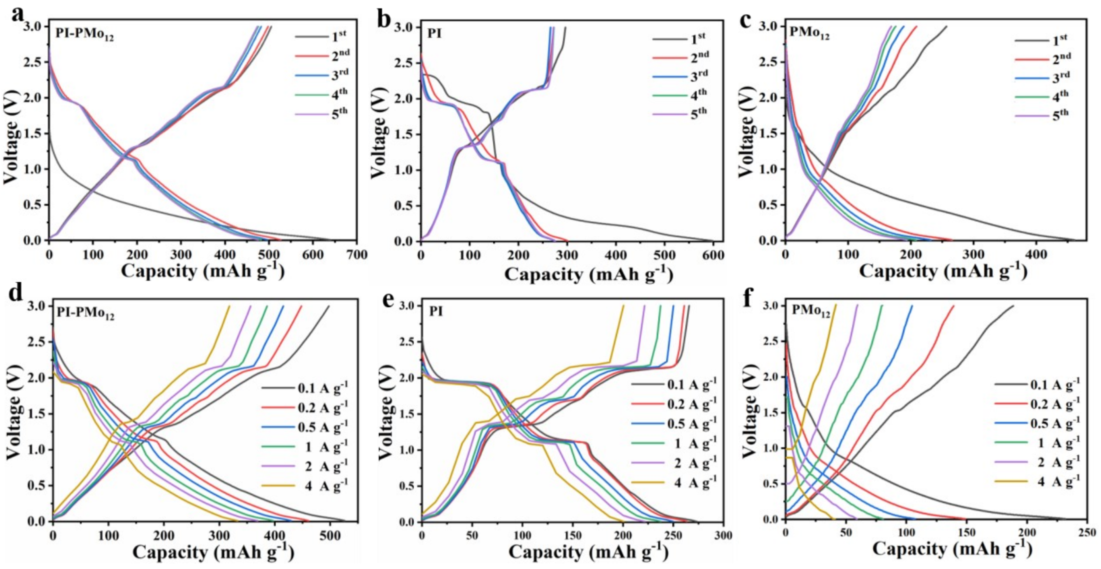

Fig. S6 GCD profiles of (a) PI-PMo_{12}, (b) PI and (c) PMo_{12} anodes at 0.1 A g^{-1}; GCD profiles at different current densities of (d) PI-PMo_{12}, (e) PI and (f) PMo_{12} anodes.

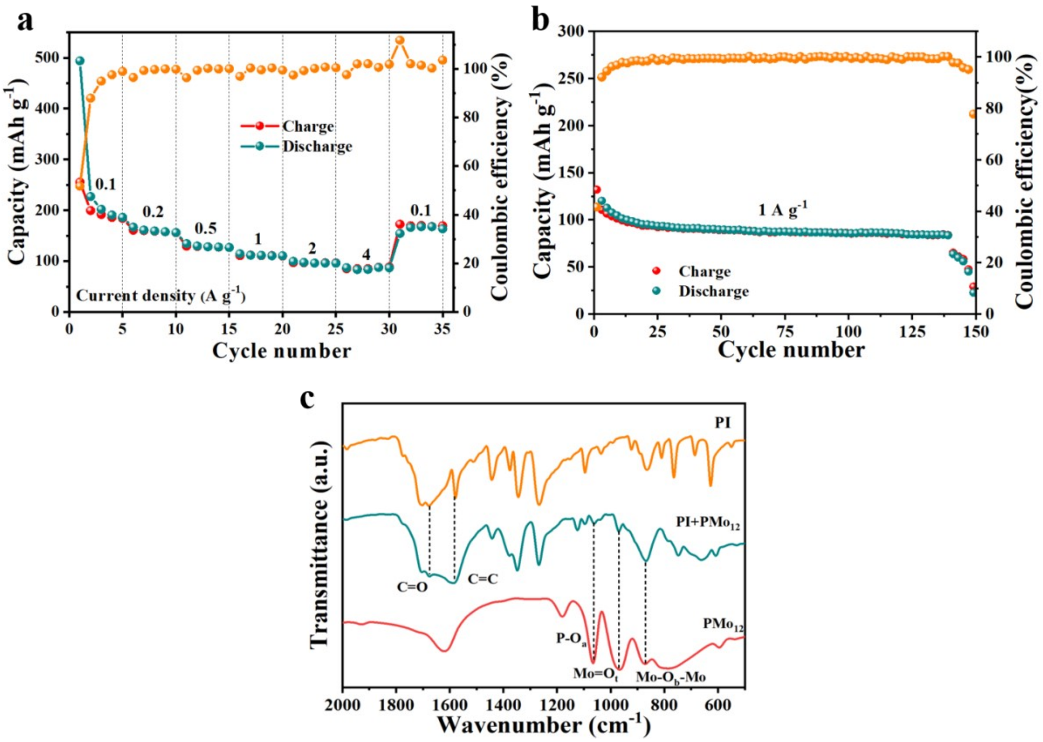

Fig. S7 (a) Rate performance, (b) cycling stability and CEs of PI+PMo_{12} anode; (c) FT-IR spectra of PI, PI+PMo_{12} and PMo_{12}.

A physically mixed control electrode (denoted as PI+PMo_{12}) with the same component ratio as the PI-PMo_{12} sample was prepared. As shown in Fig. S7a, the PI+PMo_{12} electrode delivers a low reversible capacity of only 202.1 mAh g^{-1} at a current density of 0.1 A g^{-1} and exhibits severe capacity decay after 139 cycles at 1 A g^{-1} (Fig. S7b). The FTIR spectrum (Fig. S7c) of the physically mixed sample shows a simple superposition of the characteristic peaks of pristine PI and PMo_{12}, with no peak shifts or new peaks observed.

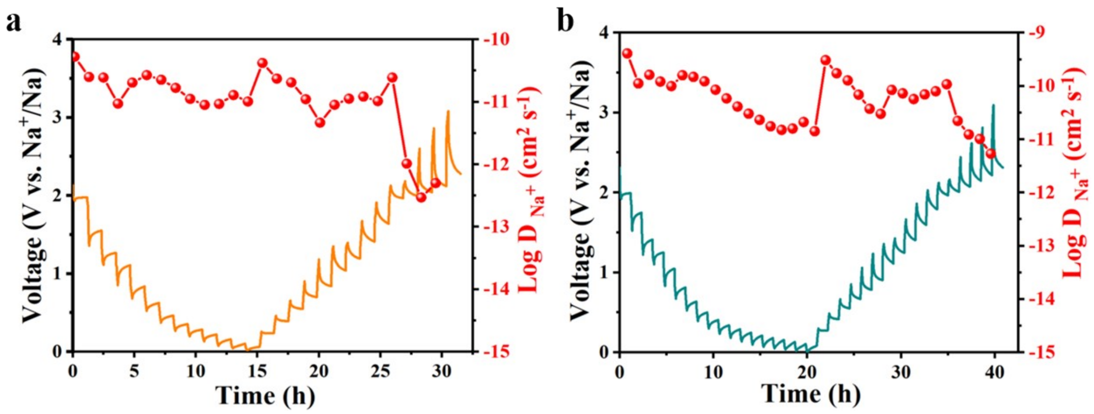

Fig. S8 GITT profiles and calculated diffusion coefficients of (a) PI and (b) PI-PMo_{12} anodes.

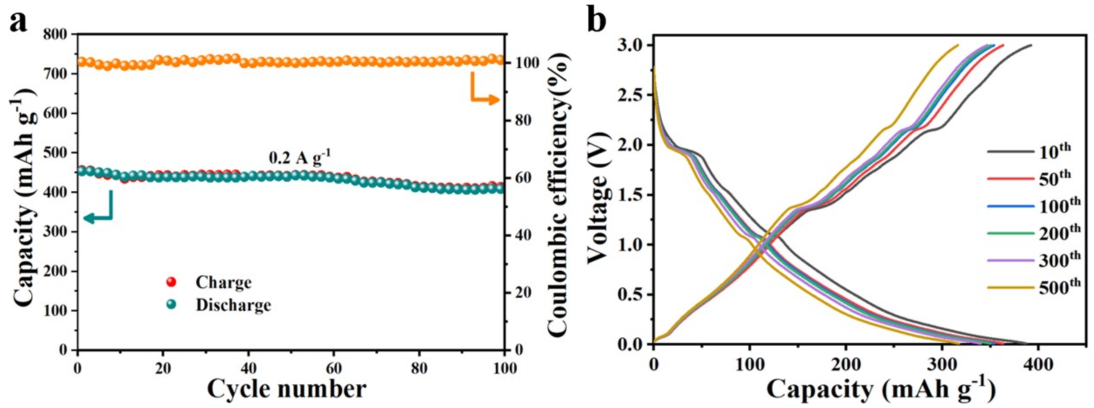

Fig. S9 (a) Cycling stability and CEs of PI-PMo_{12} anode at 0.2 A g^{-1}; (b) GCD profiles of PI-PMo_{12} at 1 A g^{-1}.

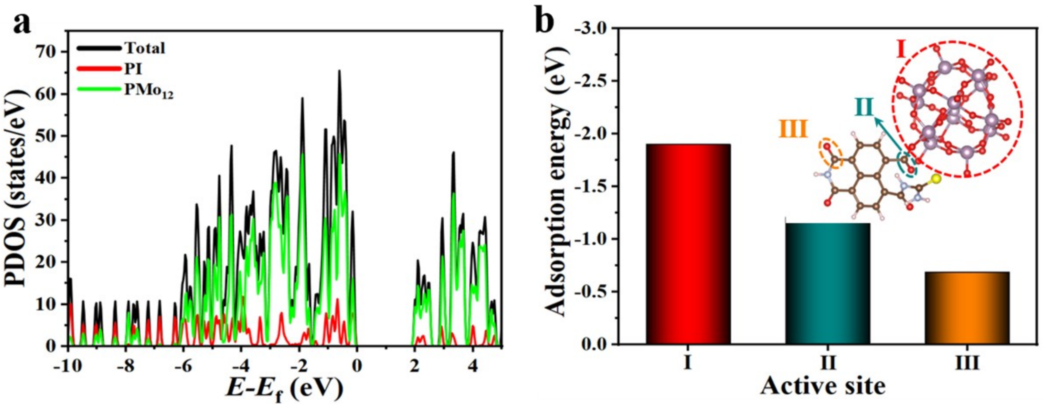

Fig. S10 (a) PDOS analysis of PI-PMo_{12}; (b) adsorption energies at different sites of PI-PMo_{12} (the inset is a structural illustration of PI-PMo_{12}).

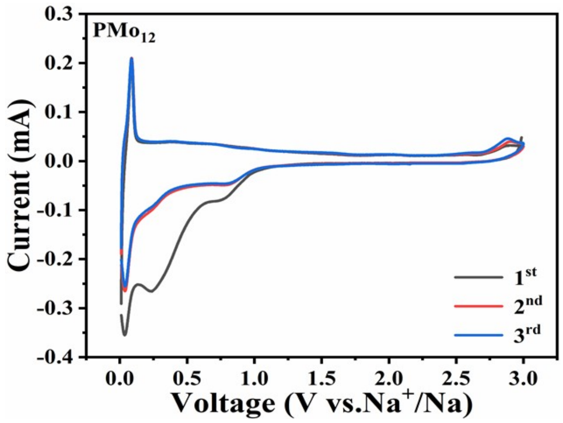

Fig. S11 CV curves of PMo_{12} anode within a potential window of 0.01-3.0 V, (scanning rate: 0.2 mV s^{-1}).

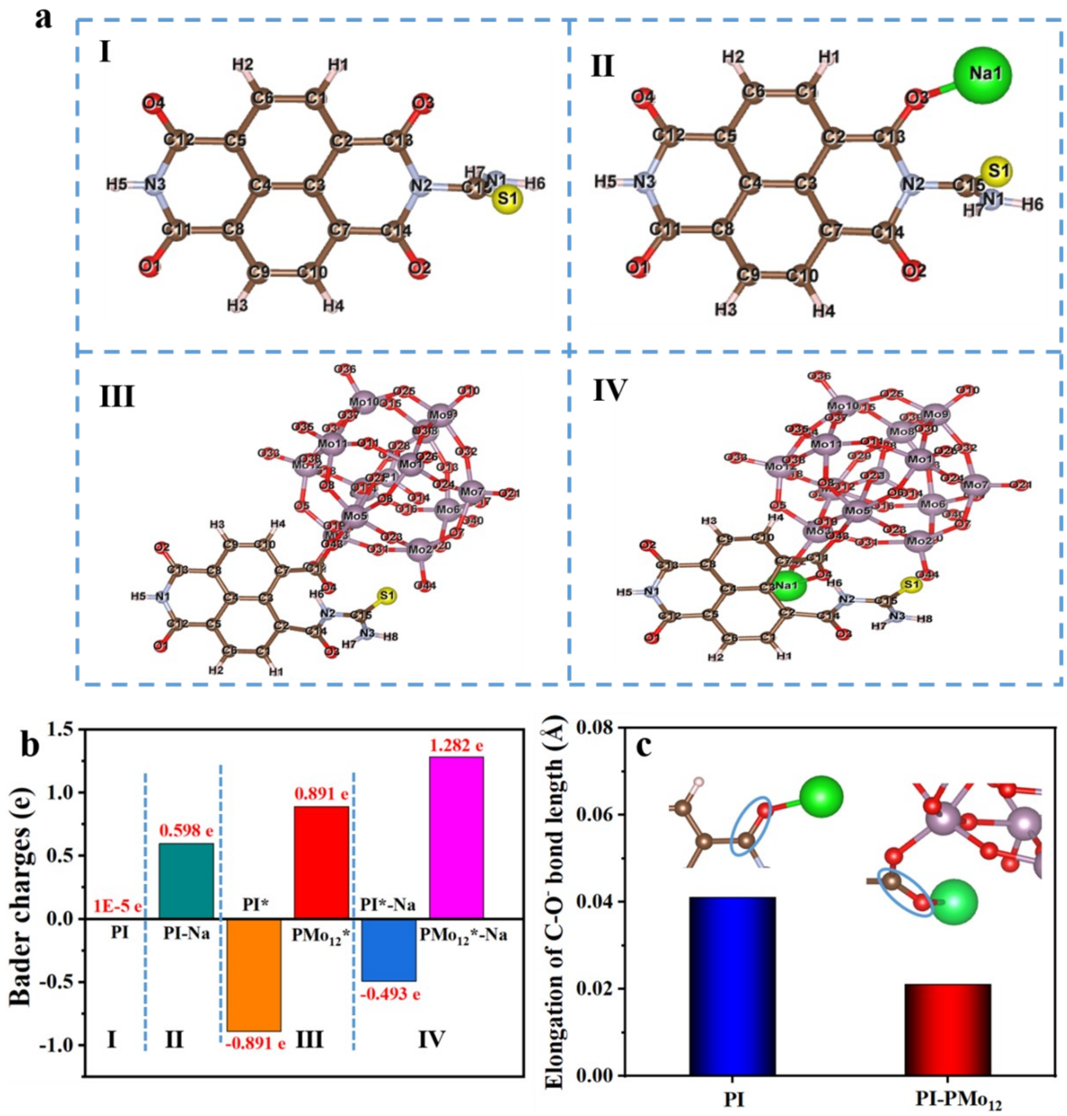

Fig. S12 (a) Structural models of PI (I), PI after sodiation (PI-Na, II), $PI-PMo_{12}$ (III) and $PI-PMo_{12}$ after sodiation ($PI-PMo_{12}$-Na, IV); (b) accumulated electron numbers of each component from Bader analysis; species with asterisks (*) are the PI and PMo_{12} components in the $PI-PMo_{12}$; the "-Na" indicates sodiated states; (c) comparison of the $C-O^-$ bond lengths elongation in PI and the $PI-PMo_{12}$ after sodiation.

Bader charge analysis reveals that pristine PI exhibits no net charge accumulation before sodiation. After sodiation, a charge accumulation of 0.598 e^- is observed on the PI framework. For the $PI-PMo_{12}$, prior to sodiation, the PI component carries a negative charge of -0.891 e^-, while the PMo_{12} component carries a positive charge of +0.891 e^-, indicating intrinsic charge transfer from PI to PMo_{12}. After sodiation, the charge on PI becomes -0.493 e^- (a net increase of +0.398 e^- relative to the pristine state), and the charge on PMo_{12} increases to +1.282 e^- (a net increase of +0.391 e^-). These results demonstrate that after sodiation, both PI and PMo_{12} in the

hybrids accumulate charge. Notably, the PI component in PI-PMo_{12} accumulates only 0.398 e^- upon sodiation, which is significantly less than the 0.598 e^- accumulated in pristine PI, corroborating that PMo_{12} effectively delocalizes excess electron density and mitigates charge accumulation on the PI backbone during discharge.

Table S1. Transferred electron numbers for PI and PI-Na derived from Bader charge analysis.

PI		PI-Na	
Atom	Electron transfer number	Atom	Electron transfer number
C1	-0.17814	C1	0.84404
C2	0.16353	C2	-0.548
C3	-0.03922	C3	0.14141
C4	-0.15508	C4	-0.76368
C5	0.11813	C5	1.15657
C6	-0.10288	C6	-0.72819
C7	0.04245	C7	-0.28045
C8	0.0654	C8	0.53559
C9	-0.13066	C9	-0.96984
C10	-0.11261	C10	-2.03436
C11	-0.98737	C11	-2.10084
C12	-1.22738	C12	0.449
C13	-1.2943	C13	-0.24295
C14	-1.11049	C14	2.66887
C15	-0.19637	C15	-0.28439
N1	0.7086	N1	-0.63096
N2	0.95781	N2	-0.5885
N3	0.82472	N3	-1.02676
H1	0.03047	H1	0.76125
H2	-0.03844	H2	0.9558
H3	0.0004	H3	0.53379
H4	-0.0254	H4	0.7825
H5	-0.33738	H5	0.52185
H6	-0.37017	H6	0.89408
H7	-0.33924	H7	0.84658
O1	0.94694	O1	1.90446
O2	0.95524	O2	-3.27248
O3	1.00295	O3	0.42668
O4	0.95904	O4	0.61913
S1	-0.13054	S1	0.02849
		Na1	-0.59866

Table S2. Transferred electron numbers for PI* and PI*-Na derived from Bader charge analysis (species with asterisks (*) are the PI components within the PI-PMo_{12} composite).

PI*		PI*-Na	
Atom	Electron transfer number	Atom	Electron transfer number
C1	-0.50996	C1	-0.49635
C2	-0.03799	C2	-0.00988
C3	-0.03276	C3	-0.01327
C4	-0.01411	C4	-0.00431
C5	-0.01367	C5	-0.00425
C6	-0.52443	C6	-0.50936
C7	-0.04092	C7	-0.01691
C8	-0.0184	C8	-0.01022
C9	-0.52204	C9	-0.51304
C10	-0.51533	C10	-0.51307
C11	-0.46775	C11	-0.46835
C12	-0.38901	C12	-0.36592
C13	-0.37001	C13	-0.37318
C14	-0.39768	C14	-0.3818
C15	-0.02639	C15	-0.0111
H1	0.47346	H1	0.47636
H2	0.46281	H2	0.46723
H3	0.4558	H3	0.45836
H4	0.48418	H4	0.47557
H5	0.37801	H5	0.38299
H6	0.49329	H6	0.47223
H7	0.37098	H7	0.37993
H8	0.37622	H8	0.38466
S1	-0.41744	S1	-0.34941
O1	0.36627	O1	0.38365
O2	0.35657	O2	0.37472
O3	0.39161	O3	0.415
O4	0.41587	O4	0.50034
N1	-0.34426	N1	-0.35387
N2	-0.34722	N2	-0.35
N3	-0.92648	N3	-0.91929
		Na1	-0.78939

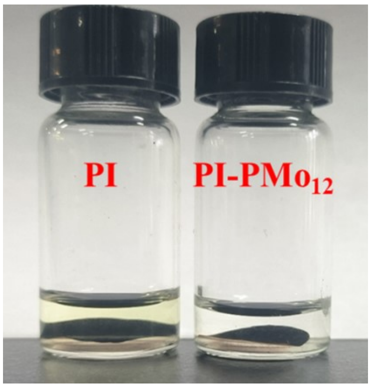

Fig. S13 Optical images of PI and PI-PMo$_{12}$ electrodes soaked in the electrolyte solvent after 200 cycles.

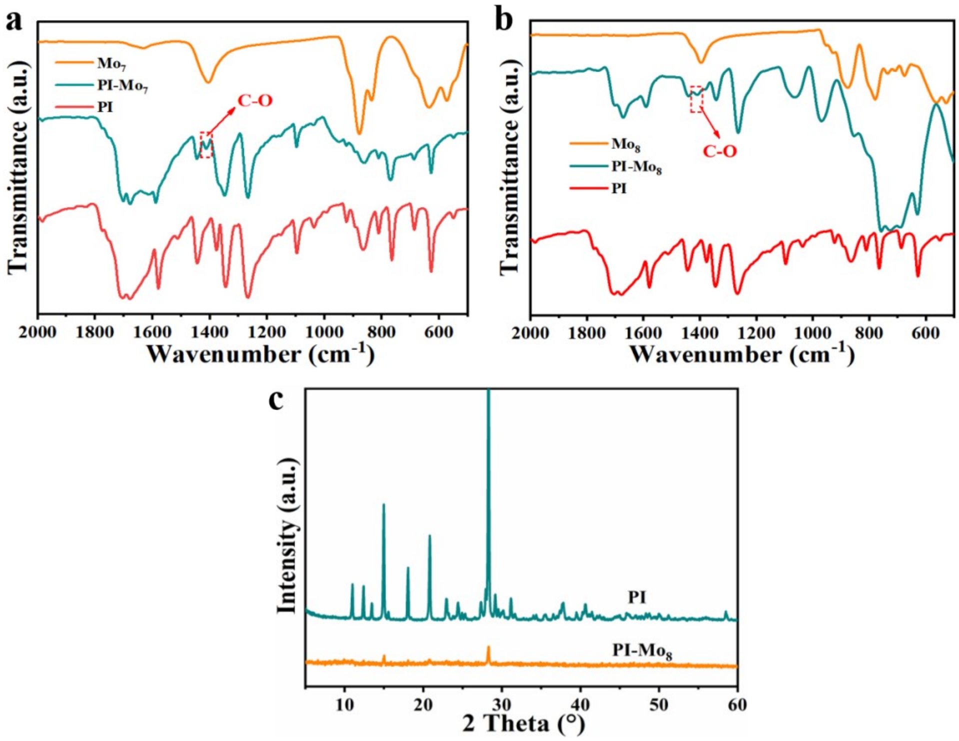

Fig. S14 (a) FT-IR spectra of Mo$_7$, PI and PI-Mo$_7$; (b) FT-IR spectra of Mo$_8$, PI and PI-Mo$_8$; (c) XRD patterns of PI and PI-Mo$_8$.

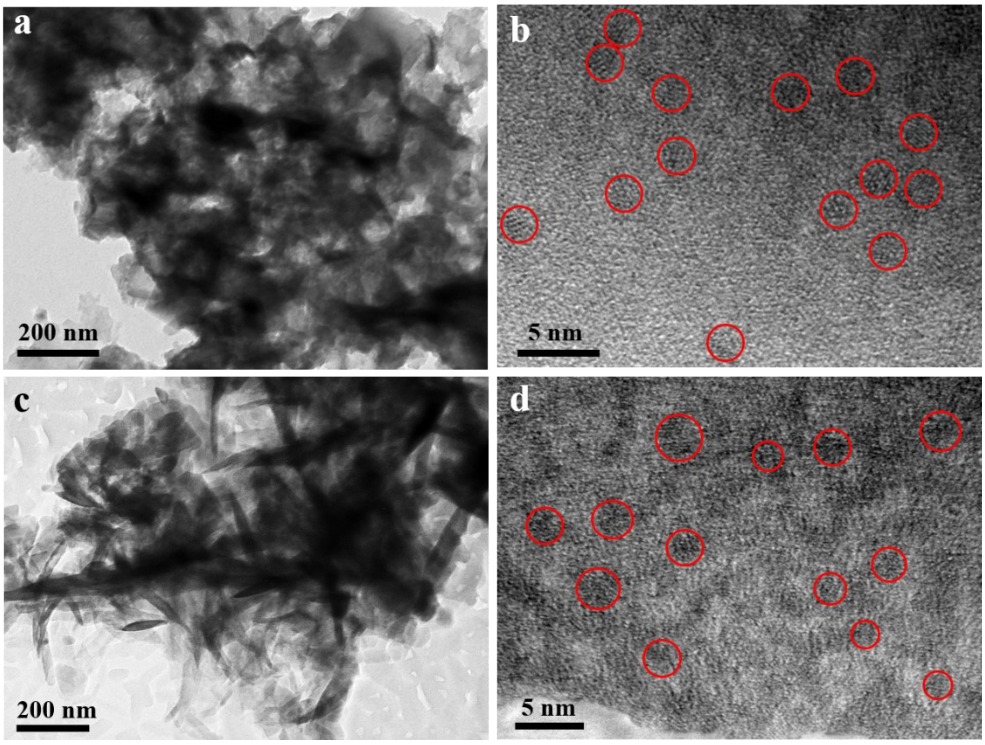

Fig. S15 (a) TEM and (b) HRTEM images of PI-Mo_7; (c)TEM and (d) HRTEM images of PI-Mo_8.

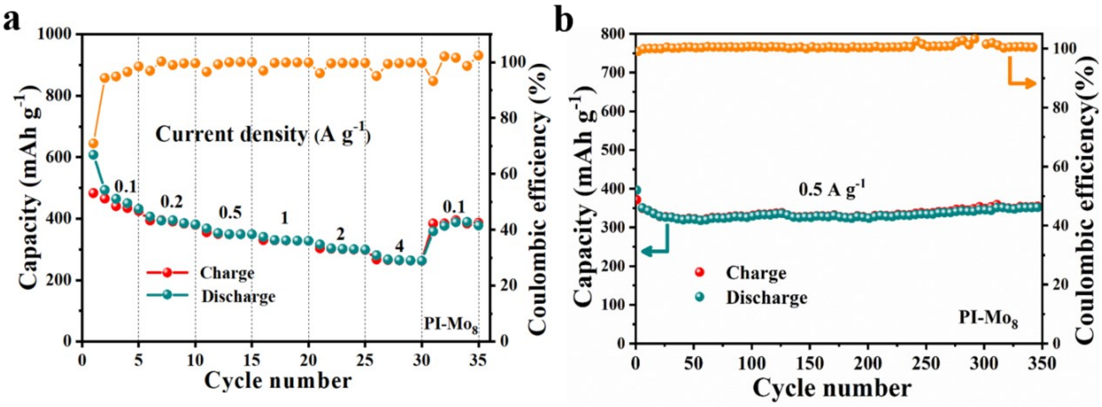

Fig. S16 (a) Rate performance, (b) cycling stability of PI-Mo_8 anode at 0.5 A g^{-1}.

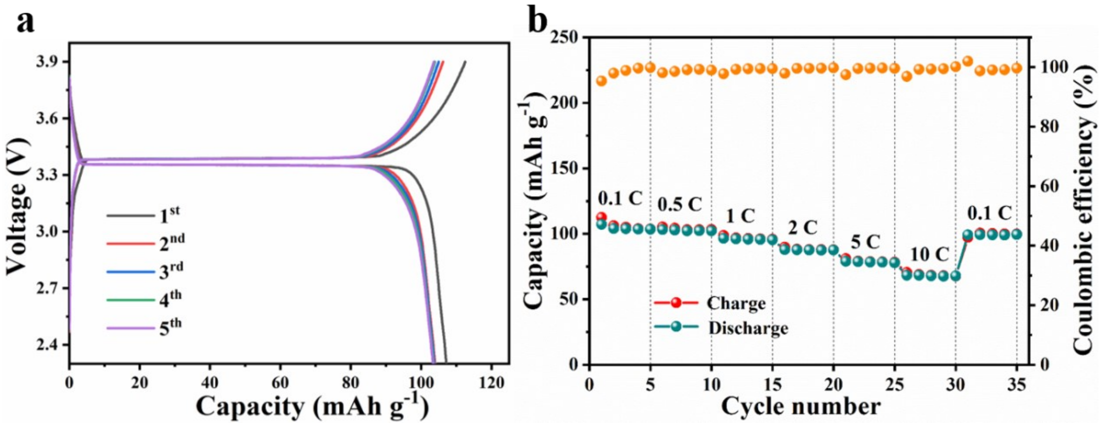

Fig. S17 (a) GCD curves of the NVP cathode at 0.1 C within a voltage window of 2.3–3.9 V; (b) rate performance of the NVP cathode.

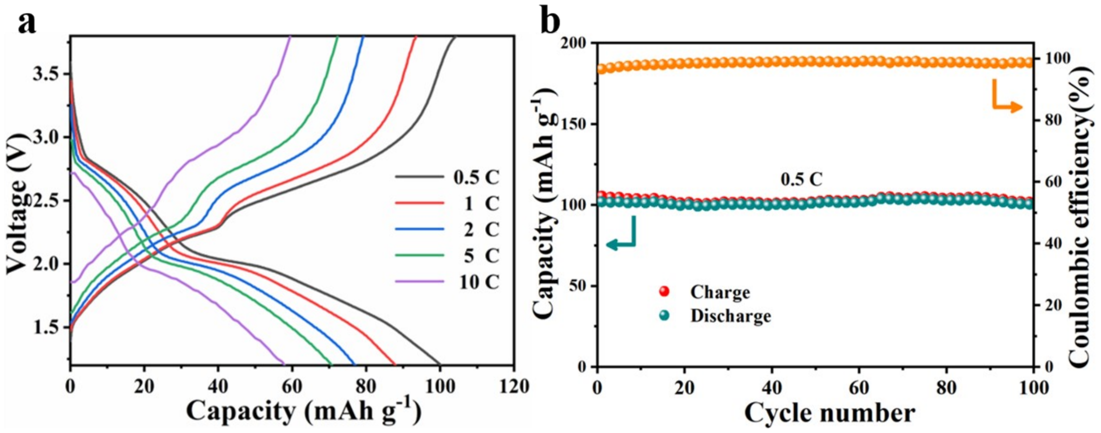

Fig. S18 (a) GCD profiles at different current densities of NVP//PI-PMo_{12}; (b) cycling stability and CEs of NVP//PI-PMo_{12} at 0.5 C.

References

[1] R. Gautam, N. Kumar, J.G. Lynam, Theoretical and experimental study of choline chloride-carboxylic acid deep eutectic solvents and their hydrogen bonds, J. Mol. Struct., 1222 (2020) 128849.

[2] A.U. Rehman, A. Maqsood, A.B. Siddique, S. Akhtar, K.F. Fawy, Q.U. Ain, M. Sher, U. Nishan, T. Ahmad, A. Ali, A. Abbas, From waste to water treatment: Banana peel powder for polystyrene removal with FTIR-based mechanistic understanding, J. Ind. Eng. Chem., 158 (2026) 597-611.

[3] J. Hu, F. Jia, Y.-F. Song, Engineering high-performance polyoxometalate/PANI/MWNTs nanocomposite anode materials for lithium ion batteries, Chem. Eng. J., 326 (2017) 273-280.

[4] R. Abazari, L. Esrafili, A. Morsali, Y. Wu, J. Gao, PMo_{12}@UiO-67 nanocomposite as a novel non-leaching catalyst with enhanced performance durability for sulfur removal from liquid fuels with exceptionally diluted oxidant, Appl. Catal. B-Environ., 283 (2021) 119582.
